# Reinvigoration of diploid strawberry (*Fragaria vesca*) during adventitious shoot regeneration

**DOI:** 10.1038/s41598-019-49391-8

**Published:** 2019-09-10

**Authors:** Hua Wang, Yuan Yang, Maofu Li, Jiashen Liu, Wanmei Jin

**Affiliations:** 1 Beijing Academy of Forestry and Pomology Sciences, Beijing Academy of Agriculture and Forestry Sciences, Beijing, 100093 China; 2 Key Laboratory of Biology and Genetic Improvement of Horticultural Crops (North China), Ministry of Agriculture, Beijing, 100093 China; 3 Beijing Engineering Research Center for Deciduous Fruit Trees, Beijing, 100093 China

**Keywords:** Tissue engineering, Plant biotechnology

## Abstract

Diploid strawberry (*Fragaria vesca* ‘Baiguo’) is a model plant for studying functional genomics in Rosaceae. Adventitious shoot regeneration is essential for functional genomics by *Agrobacterium tumefaciens*-mediated transformation. An efficient shoot regeneration method using diploid strawberry leaf explants was conducted on 1/2MS + 1/2B_5_ medium that contained 2.0 mg L^−1^ TDZ over 14 days of dark culture; this induced the maximum percentage of shoot regeneration (96.44 ± 1.60%) and the highest number of shoots per explant (23.46 ± 2.14) after 11 weeks of culture. The explants considerably enlarged after 12 days; then, turned greenish brown after 30 days, yellowish brown after 36 days, and completely brown and necrotic after 48 days. Large numbers of adventitious shoots were produced from 48 to 66 days, and the shoots elongated from 66 to 78 days; this represents a critical period of reinvigoration, which included 30 days for leaf explant chlorosis, 36 days for adventitious shoot appearance, and 48 days for generation of numerous shoots. During the reinvigoration process, higher expressions of the hormone synthesis-related genes *Ciszog1*, *CKX2*, *CKX3*, *CKX7*, *YUC2*, *YUC6*, *YUC10*, *YUC9*, and *GA2ox* were detected from 30 to 48 days. Our results indicate that these genes may regulate reinvigoration of shoot regeneration.

## Introduction

Diploid strawberry (*Fragaria vesca*), also known as the woodland strawberry, has a small genome (2n = 2× = 14, 240 Mbp), easy vegetative propagation, and a small, herbaceous stature^1^. These advantages make diploid strawberry more suitable as an attractive model than the commercial octoploid strawberry (*Fragaria* × *ananassa*) (2n = 8× = 56) and other plants of Rosaceae for functional genomics research^2^. Adventitious shoot regeneration is essential for subsequent functional genomics research by *Agrobacterium tumefaciens*-mediated transformation and is affected by internal and external influences.

In strawberry, the effects of basic medium^3^, plant growth regulators^4–6^, explant types^6,7^, cultivars^5,7^, and duration of dark culture^8^ have been demonstrated on adventitious shoot regeneration of leaf explants. In basic medium, the shoots that developed from the buds were subcultured onto 1/2 Murashige and Skoog (MS) + 1/2B_5_ + 0.2 mg L^−1^ 6-benzyladenine (BA) + 0.1 mg L^−1^ indole-3-butyric acid (IBA), MS + Fe-EDDHA (Ethylenediamine di-2-hydroxyphenyl acetate ferric)^9^ + 0.2 mg L^−1^ BA + 0.1 mg L^−1^ IBA, and 1/2MS + 0.2 mg L^−1^ BA + 0.1 mg L^−1^ IBA. Diploid strawberry grew best in 1/2MS + 1/2B_5_ + 0.2 mg L^−1^ BA + 0.1 mg L^−1^ IBA (Fig. S1). When exposed to plant growth regulator, the best shoot regeneration frequency in cultivated octoploid strawberry (*F*. *ananassa* cv. ‘Honeoye’), 94.7%, was obtained on MS medium supplemented with 2.0 mg L^−1^ thidiazuron (TDZ)^8^. TDZ + IBA promoted the highest shoot regeneration efficiencies in leaves of nearly all genotypes, whereas the TDZ/3-Benzo[b] selenienyl acetic acid (BSAA) and TDZ/2,4-Dichlorophenoxy acetic acid (2,4-D) combinations promoted high regeneration efficiencies in only some genotypes^5^. Additionally, explants kept for 14 days in dark culture showed the highest regeneration percentage of adventitious shoots in explants (100%), and produced an average of 3.6 shoots per explant in ‘Pingyitiancha’ (*Malus hupehensis* var. *pinyiensis*)^10^. Based on these results, we report an efficient protocol obtained using 1/2MS + 1/2B_5_ medium supplemented with 2.0 mg L^−1^ TDZ, 20 g L^−1^ sucrose, and 6 g L^−1^ agar (pH 5.875) for *in vitro* shoot regeneration of diploid strawberry *F*. *vesca* ‘Baiguo’.

Notably, we found that regenerating competence recovered in the nearly brownish diploid strawberry explants, and shoots were regenerated from 30 to 48 days of culture. This may represent a selective process in which cells with low competence in response to the plant growth regulator treatment are killed, whereas a few competent cells from specific leaf tissue, potentially parenchymatic cells, start to divide and differentiate into a new adventitious shoot over time^11^. Similarly, “reinvigoration” of plants has been pursued by methods such as grafting and 6-benzyladenine metabolism. The term reinvigoration refers to the reversion of senescence^12,13^ and was used to describe the reversion of senescence that occurs when leaf explants are cultured in the regeneration medium^14^. This senescence is a general response that occurs in all strawberry explants, cultivated or wild, and it is reflected by chlorosis of the explant prior to regeneration^8,15^. Reinvigoration is critical for efficient shoot regeneration in diploid strawberry. However, less is known about molecular aspects of leaf explant reinvigoration events. Molecular mechanisms underlying shoot regeneration have been extensively studied, and considerable evidence has demonstrated important roles of plant growth regulators, such as cytokinins^16^, auxin^17^, and gibberellin^18^, in regulating shoot regeneration. Consequently, the genes involved in hormonal control may affect reinvigoration of leaf explants during shoot regeneration.

In this study, we reported a reinvigoration phenomenon during adventitious shoot regeneration of diploid strawberry, analysed the detailed changes over a long period (at least 11 weeks), and carried out a detailed transcript analysis of related genes during reinvigoration. The relationships between reinvigoration of adventitious shoot regeneration and transcript level variations were also revealed.

## Results and Discussion

### Reinvigoration during adventitious shoot regeneration of diploid strawberry *F*. *vesca* ‘Baiguo’ leaf explants

**Figure 1:**
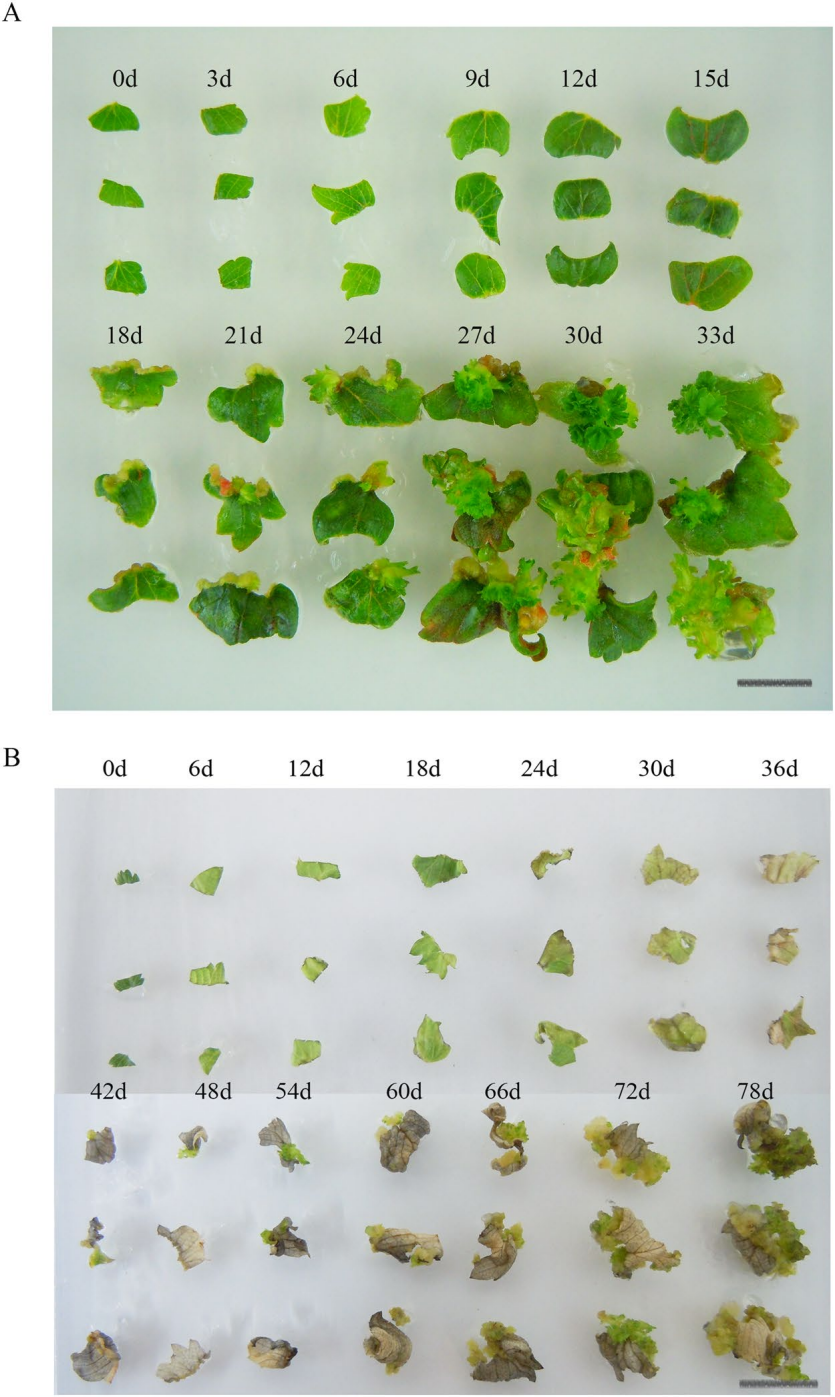
Adventitious shoot regeneration of strawberry leaf explants. (**A**) Octoploid strawberry (*F*. *ananassa* cv. ‘Honeoye’) leaf explants. (**B**) Diploid strawberry (*F*. *vesca* ‘Baiguo’) leaf explants. Note: Bar, 1 cm.

The morphogenic responses of somatic tissue have been studied *in vitro*. There are several lines of *F*. *vesca* and *F*. *ananassa* that have been tested for *in vitro* regeneration and showing a different behaviour^19^. Several blackberry and raspberry lines have been tested for *in vitro* regeneration and showed different morphogenic responses to plant growth regulator combinations^20^. Our results showed that adventitious shoot regeneration of diploid strawberry *F*. *vesca* ‘Baiguo’ differed from that of octoploid strawberry ‘Honeoye’. The octoploid strawberry leaf explants were green during regeneration (Fig. 1A). The explants showed elongation and enlargement after 6 days of culture. The shoot organogenesis from very small callus was formed from the cuts of explants after 15 days culture by histological observations^8^. A large callus formed after 18 days of culture. Relatively small shoots were observed that were sporadically dispersed within the callus on day 21 of culture, and clustered shoots were observed on day 27. The number of adventitious shoots and their growth increased over time, with most shoots being longer on day 33 of culture^8^. During whole shoot regeneration of octoploid strawberry, the leaf explants remained green and invigorated, and adventitious shoots typically appeared after 33 days.

Compared with octoploid strawberry ‘Honeoye’, adventitious shoots regenerated more in browning leaf explants of *F*. *vesca* ‘Baiguo’ (Fig. 1B). After 12 days of culture, the *F*. *vesca* ‘Baiguo’ explants considerably enlarged. After 30 days of culture, the explants turned greenish brown. After 36 days of culture, the explants turned yellowish brown, produced a yellow-green callus, and sporadically formed adventitious shoots around the wound edges. After 48 days of culture, the explants turned completely brown, but many shoots appeared. From 48 to 66 days of culture, adventitious shoots nearly doubled in number and elongated. After 66 days of culture, adventitious shoots were even more elongated. Therefore, the critical periods occurred when there was reinvigoration of completely brown explants that resulted in shoot regeneration, which included 30 days for chlorotic leaf explants, 36 days for adventitious shoot appearance, and 48 days for generation of several shoots.

### Dark treatment effects on reinvigoration during adventitious shoot regeneration from the leaf explant of *F*. *vesca*

**Figure 2:**
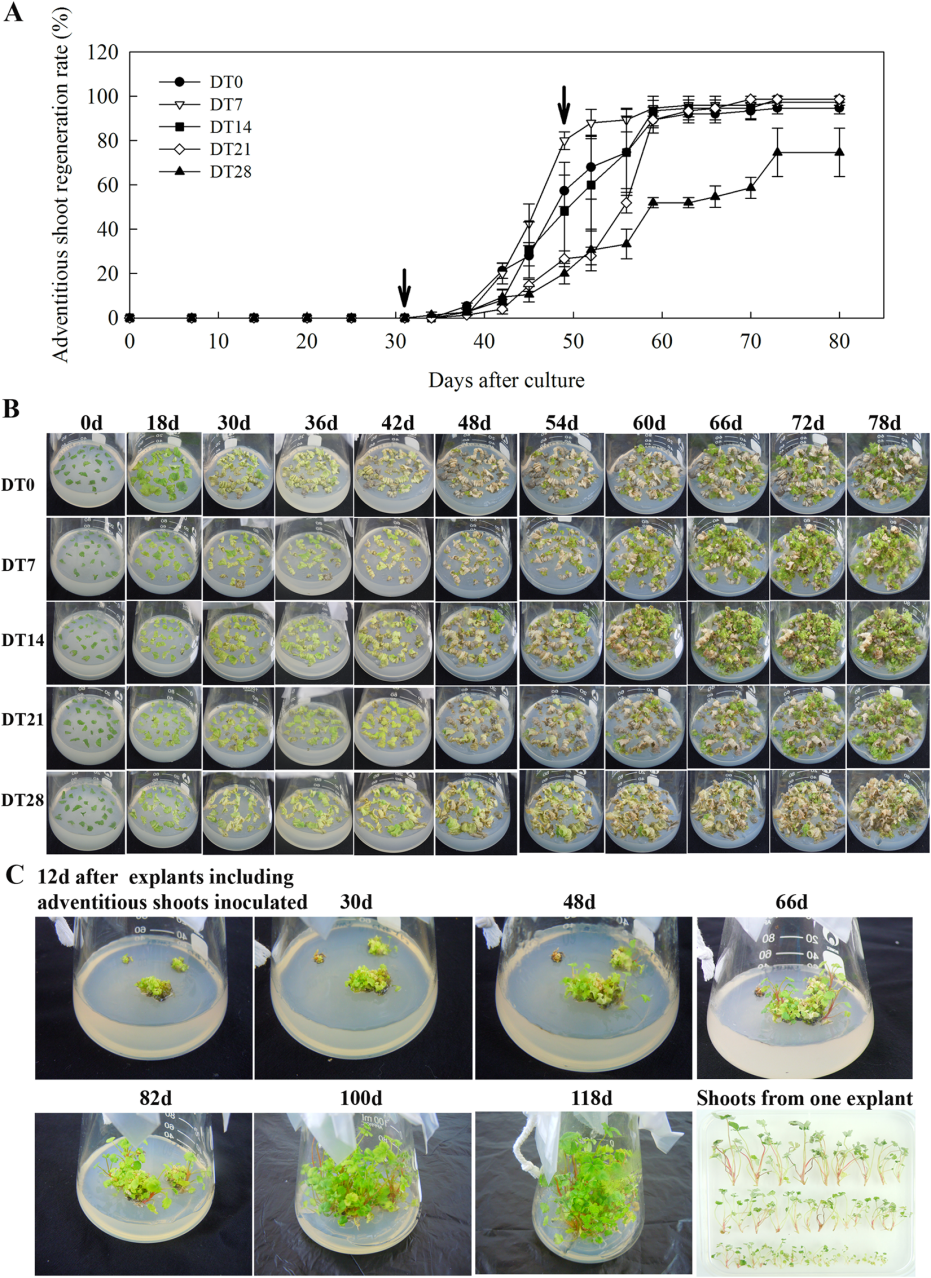
Dark treatment effects on reinvigoration during adventitious shoot regeneration of diploid strawberry leaf explants. (**A**) Adventitious shoot regeneration of diploid strawberry *F*. *vesca* ‘Baiguo’ leaf explants in the light (DT0) and in the dark for 7 days (DT7), 14 days (DT14), 21 days (DT21), or 28 days (DT28) after 78 days on 1/2MS + 1/2B_5_ medium that contained 2.0 mg L^−1^ TDZ. Arrows indicate the reinvigoration period of adventitious shoot regeneration. (**B**) Adventitious shoot regeneration on 0, 18, 30, 36, 42, 48, 54, 60, 66, 72, and 78 days of culture under different dark treatments. (**C**) Culture of the explants including adventitious shoots inoculated on 1/2MS + 1/2B_5_ medium contained 0.2 mg L^-1^ BA and 0.1 mg L^-1^ IBA.

Subsequently, to study the effect of dark treatment on reinvigoration, various periods of initial dark culture (from 0 to 28 days) were applied. Although dark treatments did not influence the reinvigoration outcomes, the duration of dark culture did (Tables 1 and S1). Both the control explants (0 days, no initial dark culture treatment) and explants initially kept under dark culture conditions regenerated adventitious shoots. The adventitious shoot regeneration rate (from 90.67 ± 7.34% to 96.44 ± 1.60%) and number of shoots per explant (from 19.62 ± 1.88 to 23.46 ± 2.14) after the initial 7, 14, and 21 days of dark culture were not different from that in the control, but were significantly higher than that during the initial 28 days of dark culture (63.56 ± 10.45% and 7.86 ± 1.52 for adventitious shoot regeneration rate and number of shoots per explant, respectively) (*p* < 0.05) (Tables 1 and S1). On the other hand, dark treatments did not influence the reinvigoration process, the duration of dark culture did (Fig. 2A). However, dark treatments only influenced reinvigoration for the first 7 days. The other dark treatments are lower than 0 day in adventitious shoot regeneration rate between 42–60 days after culture (Fig. 2A). Furthermore, chlorosis of leaf explants after the initial 7, 14, and 21 days of dark culture from 30 to 48 days was less obvious than in the control, but more obvious than that in the initial 28 days of dark culture (Figs 2B and S2); this might be attributed to light having negative interactions with hormones that induces oxidative stress which kills cells^21^, dark incubation enhancing the accumulation of endogenous IAA^22^, and dark treatments leading to the reduction of photosynthetic capacity and a reduced efficiency of the chloroplasts^8,10,23^. We also found that, when the regenerated shoots were inoculated on 1/2MS + 1/2B_5_ medium, the leaves of shoots turned yellow after 66 days; then, there was reinvigoration, which facilitated more continuous shoot growth and vigorous shoot generation after 66 days of culture (Fig. 2C). The results showed that dark treatments did not affect the leaf explant reinvigoration, but the duration of dark culture did. The best dark culture condition for reinvigoration (14 days) was then used to study the molecular aspects of reinvigoration.

**Table 1:** Adventitious shoot regeneration rate and number of shoots per explant of diploid strawberry (*F*. *vesca* ‘Baiguo’).

DT (day)	No. of explants	No. of regenerating explants	No. of adventitious shoots	Adventitious shoot regeneration rate (%)	No. of shoots per explant
0	225	188	3822	83.56 ± 13.86^ab^	16.99 ± 3.59^a^
7	225	204	4732	90.67 ± 7.34^ab^	21.03 ± 4.14^a^
14	225	217	5279	96.44 ± 1.60^a^	23.46 ± 2.14^a^
21	225	212	4415	94.22 ± 3.80^a^	19.62 ± 1.88^a^
28	225	143	1768	63.56 ± 10.45^b^	7.86 ± 1.52^b^

Values represent means ± standard error. Letters indicate statistically significant differences within treatments (Duncan’s significant difference test, *p* < 0.05).

### The relation between transcript level of hormone synthesis related genes and morphological characteristics such as explant reinvigoration, shoot regeneration of the leaf explant of ‘*F*. *vesca*’

**Figure 3:**
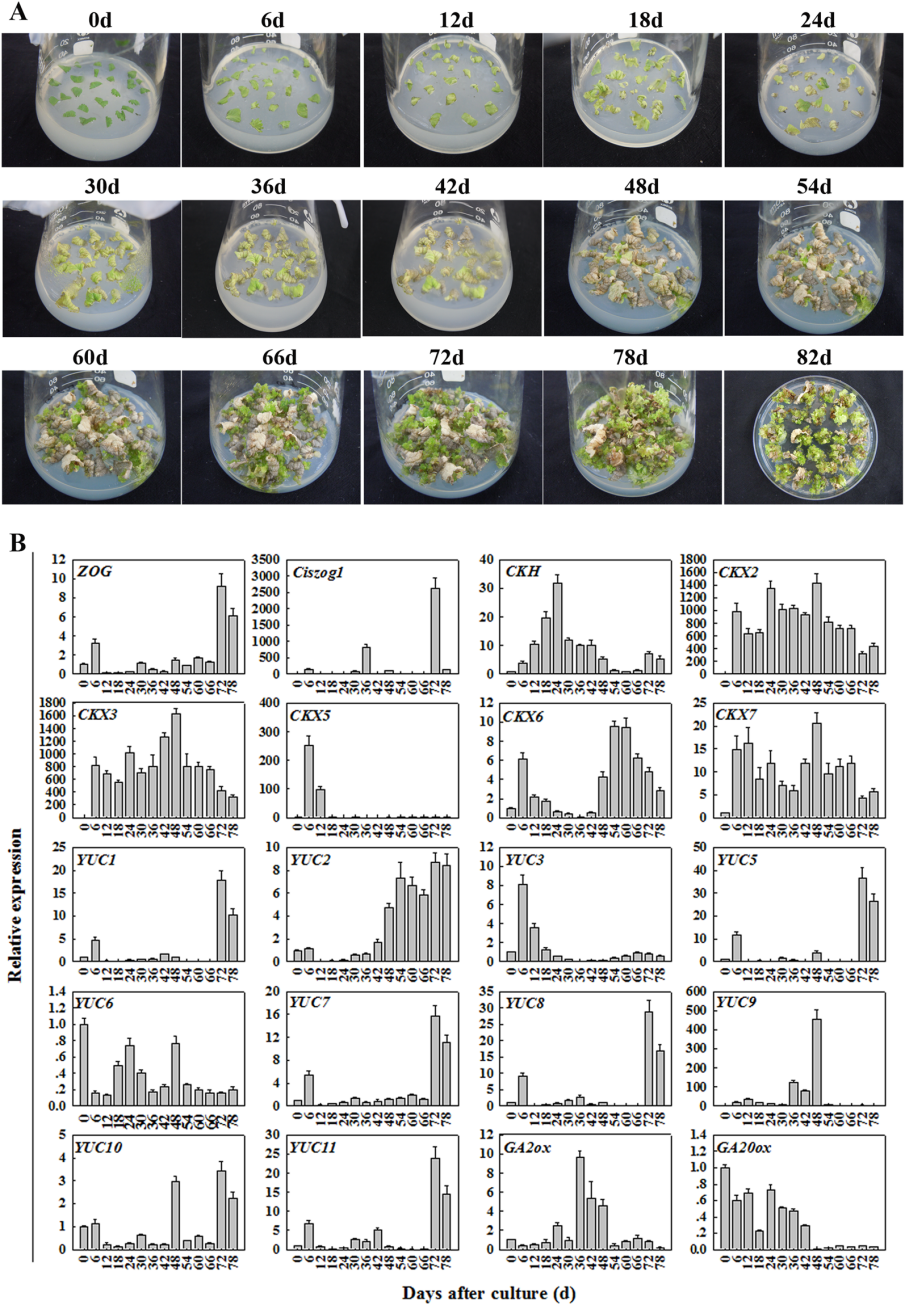
Relationships between hormone transcript levels and morphological characteristics (explant reinvigoration and shoot regeneration). (**A**) Morphological characteristics of leaf explants in initial 14 days dark treatment during shoot regeneration. (**B**) Transcript levels of key hormone synthesis-related genes, including Zeatin O-glucosyltransferase (*ZOG*), cis-zeatin O-glucosyltransferase (*Ciszog*), cytokinin hydroxylase (*CKH*), cytokinin oxidase/dehydrogenase (*CKX*) families, YUCCA (*YUC*) families, gibberellin 2-beta-dioxygenase (*GA2ox*), and *GA20ox* during diploid strawberry ‘Baiguo’ shoot regeneration.

Cytokinins play important roles in plant growth and development. Zeatin O-glucosyltransferase (*ZOG*) and cis-zeatin O-glucosyltransferase (*Ciszog*) are very important in cytokinin homeostasis^24^. The *ZOG* and *Ciszog* transcript levels were relatively higher at 6, 30, and 36 days of culture, during which leaf explants began to enlarge or produce shoot primordia (Fig. 3A,B). This is consistent with zeatin concentration variation in *in vitro*-cultivated strawberry (*F*.*ananassa* cv. ‘Honeoye’) leaf explants; the zeatin concentrations peaked at 5.98 ng g^−1^ Fw during meristemoid formation and reached 7.69 ng g^−1^ Fw during shoot primordia formation^8^. Furthermore, the *ZOG* and *Ciszog* transcript levels were the highest at 72 days of culture, during which senescence was delayed and adventitious shoots very elongated. This could be explained by the two-step adventitious shoot regeneration process that was first observed in *in vitro*-derived lingonberry leaves^25^.

Cytokinin hydroxylase (*CKH*) is involved in trans-zeatin biosynthesis, and contributes to tissue and organ formation^26^. The *CKH* transcript level significantly increased from 0 to 24 days of culture, which was during leaf explant shoot primordia formation. The *CKH* transcript level was higher from 30 to 48 days of culture, during which reinvigoration occurred and leaf explants began to produce multiple shoots (Fig. 3A,B).

Cytokinin oxidase/dehydrogenase (*CKX*) is involved in cytokinin degradation and plays important roles in plant meristem activity and morphogenesis, including causing changes in root growth and xylem differentiation^27^, and has opposite regulatory functions in root and shoot meristems^28,29^. The transcript levels of some CKX genes (*CKX2*, *CKX3*, *CKX5*, *CKX6*, and *CKX7*) increased during the initial days of culture (from 0 to 6 days), which was when there was leaf explant enlargement (Fig. 3A,B). The *CKX2*, *CKX3*, and *CKX7* transcript levels significantly increased from 30 to 48 days of culture, during which leaf explants were reinvigorated and many shoots appeared. However, the *CKX2* and *CKX3* levels were lower at 12 and 18 days than at 6, 24, 30, and 36 days. Additionally, the *CKX7* level was substantially lower at 30 and 36 days compared with 12, 42, and 48 days. These peaks were consistent with the findings of zeatin concentration variation in *in vitro*-cultivated strawberry leaf explants^8^.

Noticeable increases in the transcript levels of cytokinin biosynthesis-related genes were associated with the critical periods, including the first 6 days, during which there was explant enlargement, and 30 to 48 days of culture, during which there was diploid strawberry explant reinvigoration. The results obtained are in consistent with the initial phase of leaf senescence due to cytokinin degradation by CKX.

YUCCA (*YUC*) catalyses hydroxylation of the tryptamine amino group, which is a rate-limiting step in auxin biosynthesis^30,31^. The *YUC* genes play essential roles in auxin biosynthesis and plant development, including floral organ and vascular tissue formation in *Arabidopsis*^32^, crown root development control in rice^33^, and soybean growth and development alteration^34^. The *YUC1*, *YUC2*, *YUC3*, *YUC5*, *YUC7*, *YUC8*, *YUC9*, *YUC10*, and *YUC11* transcript levels significantly increased from 0 to 6 days of culture, which was when there was leaf explant enlargement. This can be explained by dark incubation having a positive effect on auxin-related gene stimulation, and thus endogenous auxin production and eventually leaf explant enlargement^22,35^. The transcript level of *YUC6* is higher in 0 day than in 6 days may be ascribed to wounding first triggers short term wound signaling that lasts from seconds to hours, then the wound signal spreads very rapidly from the wound site to mesophyll cells and activates *YUC* expression in mesophyll cells^36^. The *YUC2*, *YUC5*, *YUC6*, *YUC9*, and *YUC10* transcript levels significantly increased during reinvigoration (Fig. 3A,B). These noticeable increases in *YUC* gene transcript levels during the critical periods (including 30, 36, and 48 days of culture) indicate that they have roles in diploid strawberry reinvigoration.

Gibberellin 2-beta-dioxygenase (*GA2ox*) is involved in GA inactivation, which is associated with *Glycine max* development^37^, might play critical roles in cell division^38^. The *GA2ox* transcript level was substantially higher from 30 to 48 days of culture, which was when explant reinvigoration was observed (Fig. 3A,B). *GA20ox* might modify cell growth and development associated with explant reinvigoration. The *GA20ox* transcript level was much higher before 48 days of culture, which indicates that it is related to explant reinvigoration (Fig. 3A,B). The increased *GA2ox* levels prior to shoot regeneration, concomitant with a reduction in GA20oxydase (involved in GA synthesis), might indicate that a reduction in GAs preceded adventitious shoot formation.

Briefly, we report an efficient protocol that used 1/2MS + 1/2B_5_ medium supplemented with 2.0 mg L^−1^ TDZ, 20 g L^−1^ sucrose, and 6 g L^−1^ agar (pH 5.875) for *in vitro* shoot regeneration of diploid strawberry. During adventitious shoot regeneration, the obvious reinvigoration of nearly brownish diploid strawberry explants aroused our attention. The transcript level changes of shoot regeneration-related genes may provide a possible explanation of such reinvigoration. Plant hormone synthesis-related genes play essential roles in plant development. Higher expressions of the hormone synthesis-related genes *Ciszog1*, *CKX2*, *CKX3*, *CKX7*, *YUC2*, *YUC6*, *YUC10*, *YUC9*, and *GA2ox* were detected from 30 to 48 days of culture during leaf explant reinvigoration. Thus, we conclude that efficient recovery of nearly brownish diploid strawberry explants is closely associated with hormone synthesis-related genes.

## Methods

### Plant materials

Plant materials of diploid strawberry (*F*.*vesca*) and octoploid strawberry (*F*. *ananassa*) were obtained from the Beijing Academy of Forestry and Pomology Sciences, Beijing, China. The protocols for sterilisation and sample preparation were optimised according to the protocol described by Jin *et al*., 2014 and Wang *et al*.^8,10^. The disinfected lateral and apical buds were then placed on MS medium that contained 0.5 mg L^−1^ BA and 0.1 mg L^−1^ IBA. After 30 days, the shoots that developed from the buds were subcultured onto 1/2MS + 1/2B_5_ medium that contained 0.2 mg L^−1^ BA and 0.1 mg L^−1^ IBA. The basic 1/2MS + 1/2B_5_ medium contained 20 g L^−1^ sucrose and 6.0 g L^−1^ agar (Shishi Co., Fujian, China). The pH was adjusted to 5.875 and media were autoclaved for 20 min at 121 °C. Cultures were maintained in a growth room at 25 ± 2 °C under a 16-hour light photoperiod at approximately 25 µmol m^−2^s^−1^ supplied by cool-white fluorescent lamps. The strawberry plantlets were used as experimental materials for adventitious shoot regeneration analysis.

### Reinvigoration during adventitious shoot regeneration of diploid strawberry (*F*. *versa*)

The protocol for adventitious shoot regeneration of diploid strawberry *F*. *versa* was optimised as described by Jin *et al*., 2014 and Wang *et al*. 2015^8,10^. 1/2MS + 1/2B_5_ medium supplemented with 2.0 mg L^−1^ TDZ, 20 g L^−1^ sucrose, and 6 g L^−1^ agar (pH 5.875) was applied for *in vitro* analysis of *F*. *vesca* adventitious shoot regeneration. During this process, the explants were reinvigorated from being completely brown to shoot regeneration.

Subsequently, to study the effects of an initial dark period on reinvigoration, the explants were kept in the dark for 0, 7, 14, 21, and 28 days under the growth conditions described in “Plant materials”. All dark treatments (DT0:78 days at 16-h light photoperiod; DT7:7 days at dark and 71 at 16-h light photoperiod; DT14:14 days at dark and 64 at 16-h light photoperiod; DT21: 21 days at dark and 57 at 16-h light photoperiod; DT28: 28 days at dark and 50 at 16-h light photoperiod) tested for shoot induction were analysed by preparing three subsequent repetitions of three 100 mL glass flasks (90 mm in diameter with 40 mL of medium added), each of which contained 25 segmented leaf explants. The adventitious shoot regeneration rate (%) and number of regenerated shoots per explant were calculated for each glass flask. The morphogenic responses of segmented leaf explants were observed and photographed every 6 days. The adventitious shoot regeneration rates were also recorded. There were three repetitions of each dark duration treatment (225 leaf explants for each dark treatment), and the experiment was repeated three times (repetition1: from 12, Feb. to 30, Apr., 2015; repetition 2: from 12, Jun. to 30, Aug., 2015; repetition 3: from 18, Mar. to 5, Jun., 2016).

Finally, the best regeneration condition was determined (initial 0, 7, 14, 21, or 28 days of dark culture) and applied to prepare leaf explants for transcript-level analysis of related genes at 0, 6, 12, 18, 24, 30, 36, 42, 48, 54, 60, 66, 72, and 78 days of culture. In this study, the best regeneration conditions were observed in the initial 14 days of dark culture, then 0, 6, 12, 18, 24, 30, 36, 42, 48, 54, 60, 66, 72 and 78 days of culture would be as follows: 0:0 days at dark; 6:6 days at dark; 12:12 days at dark; 18:14 days at dark and 4 days at 16-h light photoperiod; 24:14 days at dark and 10 days at 16-h light photoperiod; 30:14 days at dark and 16 days at 16-h light photoperiod; 36:14 days at dark and 22 days at 16-h light photoperiod; 42:14 days at dark and 28 days at 16-h light photoperiod; 48:14 days at dark and 34 days at 16-h light photoperiod; 54:14 days at dark and 40 days at 16-h light photoperiod; 60:14 days at dark and 46 days at 16-h light photoperiod; 66:14 days at dark and 52 days at 16-h light photoperiod; 72:14 days at dark and 58 days at 16-h light photoperiod; 78:14 days at dark and 64 days at 16-h light photoperiod. The leaf explants in the initial 14 days of dark treatment were then sampled every 6 days from 0 to 78 days (0, 6, 12, 18, 24, 30, 36, 42, 48, 54, 60, 66, 72, and 78 days) to conduct the following transcript level analysis of related genes.

The adventitious shoot regeneration rate (%) was calculated as the total number of regenerating explants divided by total number of explants after 78 days of culture and then multiplied by 100%. The number of regenerated shoots per explant was calculated as the total number of adventitious shoots divided by total number of regenerating explants after 78 days of culture.

### Transcript analysis of related genes during reinvigoration

Total RNA was extracted, and first-strand cDNA was synthesised from leaf discs every 6 days from 0 to 78 days during *F*. *vesca* shoot regeneration, as described by Jin *et al*.^39^. To further investigate the transcript levels of hormone synthesis-genes, including the YUC and CKX families, specific primers were designed with Primer 5.0 (Premier Biosoft International, Palo Alto, CA, USA) (Table 2) and acquired from Sangon Inc. (Shanghai, China). qRT-PCR was conducted with on a Bio-Rad CFX96 Real-Time PCR Detection System (Bio-Rad, Hercules, CA, USA). For qRT-PCR, the 10-μL reaction mixture included 5 μL 2 × SYBR Premix (Bio-Rad, Hercules, CA, USA), 1 μL forward primer (10 μM), 1 μL reverse primer (10 μM), 1 μL cDNA template (20 ng), and 2 μL ddH_2_O; the PCR conditions were as follows: 1 cycle at 95 °C for 30 s, 40 cycles at 95 °C for 5 s, and 1 cycle at 60 °C for 30 s. Finally, the transcription levels of different genes were analysed using the 2^−ΔΔCT^ method^40^. All analyses were performed with three biological replicates.

**Table 2:** The sequences of the oligonucleotide primers for qRT-PCR analysis were used in this study.

Gene	Accession	Primer Sequence (5′ → 3′)	Function	Reference
*Actin*	AB116565	F: GGGTTTGCTGGAGATGAT R: CATCCCAGTTGCTCACAATA	Actin	NCBI database
*Fvzog*	XM_004303598	F: CACGTTTGAAGCCACCACACATCT R: AGGCGGAGACCGAGTGGAATGT	Cytokinin biosynthesis	NCBI database
*Fvciszog*	AF318075	F: AGGGCAGCAAGCAGCGTTTCG R: TGCATGGGCCAGGCAAGCAC	Cytokinin biosynthesis	NCBI database
*FvCKH*	XM_004304085	F: GGGATGTTGCTAAATGAGAT R: AAGATGGGTTGCTGGCTAAT	Cytokinin biosynthesis	NCBI database
*FvCKX2*	XM_004288903	F: CTTTCCGTTATGGTCCTCAG R: ACTTCCCTGCTTTCCCTTGC	Cytokinin biosynthesis	NCBI database
*FvCKX3*	XM_004288904	F: TATTCCAAAGAGTCGCATCG R: TCTTCTTCGGGTACAACTGC	Cytokinin biosynthesis	NCBI database
*FvCKX5*	XM_004291784	F: GCAACGCTGGAGCATGGACT R: CTGGTTCAAGGGCAATTCTAG	Cytokinin biosynthesis	NCBI database
*FvCKX6*	XM_004303024	F: CCTGGACAAGACTGACATAA R: TGGTATAAGAAGATTGAGCC	Cytokinin biosynthesis	NCBI database
*FvCKX7*	XM_004303417	F: CATTGTGGCGTTGTTGAGGT R: AAGTGCCGCTTCCATTCCTC	Cytokinin biosynthesis	NCBI database
*FvYUC1*	JF922292	F: TACCCTACATGCCTTTCCCTG R: CTTTGCGTAGTAACTTTCTTGT	Auxin biosynthesis	NCBI database
*FvYUC2*	JF922287	F: CAGATTTCCCAACCTACCCA R: GACGATTAGCCACTGACAAAC	Auxin biosynthesis	NCBI database
*FvYUC3*	JF922289	F: TGATACTCGGCAGCATTGAG R: TGATTCCGTGGACTACTTTGAT	Auxin biosynthesis	NCBI database
*FvYUC5*	JX417079	F: TGGAGGAAGTAGAGGTAGTCA R: AAGGGCTTGTAGGGTAGTGC	Auxin biosynthesis	NCBI database
*FvYUC6*	HQ848323	F: GGCTGAAGGAGGGAGGGATG R: GCGACCGTGTAAAGGGAGTAG	Auxin biosynthesis	NCBI database
*FvYUC7*	JF922288	F: GCCGCTTTGGCTTGCTGACA R: AACCACCTTGATTTCACCAG	Auxin biosynthesis	NCBI database
*FvYUC8*	NM_001280034	F: ATGGAGGAAGTAGATGTAGTCA R: CAGGGAAAGGCATGTAGGGT	Auxin biosynthesis	NCBI database
*AtYUC9*	NM_100299	F: AAGGAGTCCCATTCGTTGTG R: TTTCGTTGGGTATTCAGGGT	Auxin biosynthesis	^41^
*FvYUC10*	JF922291	F: GTGATAATAGTCGGTGCTGG R: ACATAAGTGGGACAAGAGGAC	Auxin biosynthesis	NCBI database
*FvYUC11*	JF922290	F: TGGGTTCCCTCCAAGTGTAT R: TTGCCACAACCAACAACTAACA	Auxin biosynthesis	NCBI database
*FvGA2ox*	XM_004308891	F: CAAATAAGGAGTGGTCGGAAGT R: AAAGAGGGCAGATTGTAGTGTC	GA biosynthesis	NCBI database
*FvGA20ox*	DQ195504	F: GAAGTGTTTGTGGACGAAGAA R: CGAACTATCAATCAACTCCCTC	GA biosynthesis	^42^

Note: F, forward primer; R, reverse primer.

### Statistical analysis

The results shown are the mean values of three independent experiments. In each independent experiment, all dark treatments (75 leaf explants for each treatment) tested for shoot induction were analysed by three repetitions of three 100 mL glass flasks, each of which contained 25 segmented leaf explants. Statistical analyses were performed in SPSS 16.0 (SPSS Inc., Chicago, IL, USA). One-way ANOVA was applied for parameters (adventitious shoot regeneration rate, number of shoots per explant) to evaluate the effect of dark treatment. The significance of differences was determined according to Duncan’s multiple range tests. Differences at *p* < 0.05 were considered significant.

## Supplementary information

Supplementary Information
