# Supplementary material for: Reinvigoration of diploid strawberry (Fragaria vesca) during adventitious shoot regeneration
Source: Sci Rep. 2019 Sep 10;9:13007. doi: 10.1038/s41598-019-49391-8 (PMC6736952; doi:10.1038/s41598-019-49391-8)
Supplement: Supplementary file 1 — Supplementary Information [file 41598_2019_49391_MOESM1_ESM.pdf]

Supplementary information for

Reinvigoration of diploid strawberry (*Fragaria vesca*) during adventitious shoot regeneration

Hua Wang, Yuan Yang, Maofu Li, Jiashen Liu & Wanmei Jin

Figure S1-2

Table S1

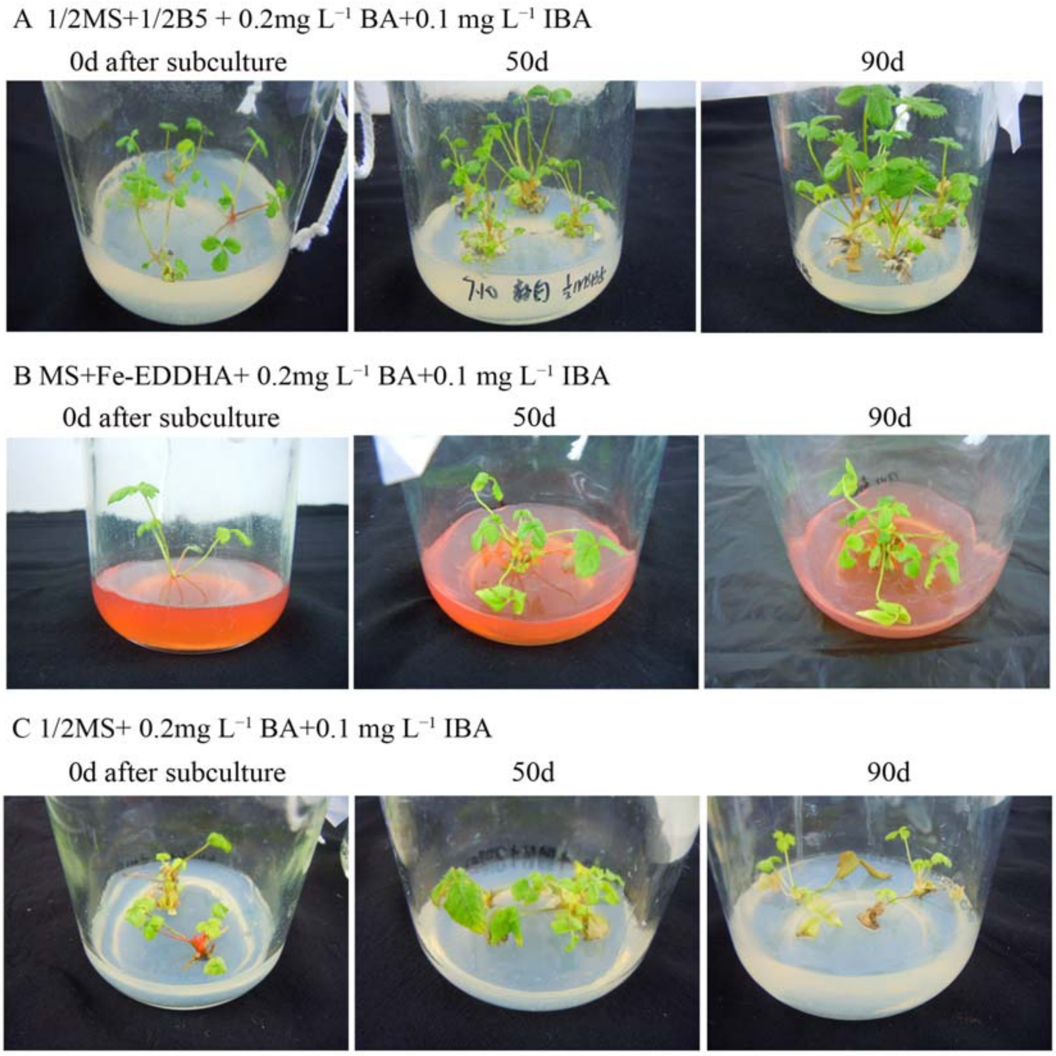

Figure S1. The shoots that developed from the buds were subcultured onto three basic mediums. (A) The shoots were subcultured onto 1/2MS+1/2B_5 + 0.2mg L^{-1} BA+0.1 mg L^{-1} IBA. (B) The shoots were subcultured onto MS+ Fe-EDDHA + 0.2mg L^{-1} BA+0.1 mg L^{-1} IBA. (C) The shoots were subcultured onto 1/2MS+ 0.2mg L^{-1} BA+0.1 mg L^{-1} IBA.

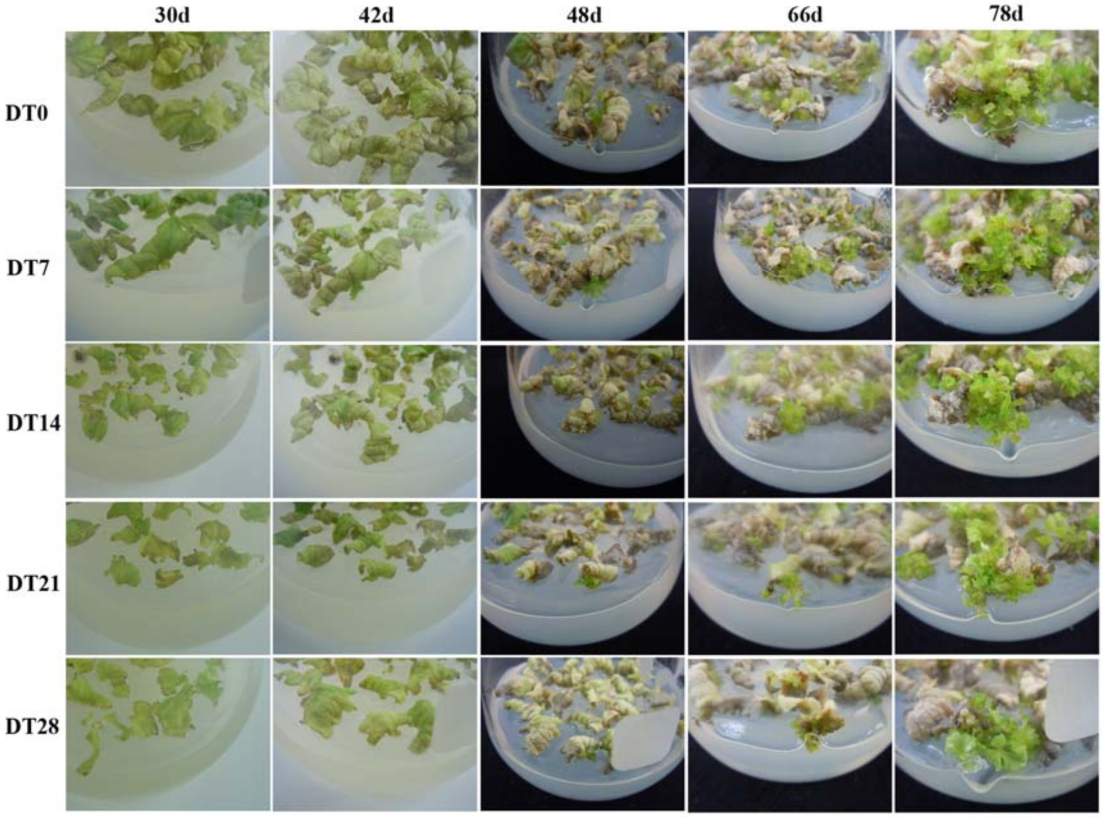

Figure S2. Dark treatment effects on reinvigoration during adventitious shoot regeneration from the leaf explant of diploid strawberry.

Table S1. Adventitious shoot regeneration rate and number of shoots per explant of diploid strawberry (*F. vesca* 'Baiguo').

Repetition	DT (day)	No. of explants	No. of regenerating explants	No. of adventitious shoots	Adventitious shoot regeneration rate (%)	No. of shoots per explant
1	0	25	23	427	94.67 ± 2.67 [a]	20.39 ±1.86 [b]
		25	25	588		
		25	23	514		
	7	25	25	697	97.33 ± 1.33 [a]	25.72 ± 1.70 [a]
		25	24	559		
		25	24	673		
	14	25	24	693	98.67 ± 1.33 [a]	27.56 ± 1.27 [a]
		25	25	742		
		25	25	632		
	21	25	24	637	98.67 ± 1.33 [a]	23.29 ± 1.35 [ab]
		25	25	521		
		25	25	589		
	28	25	17	175	74.67 ± 10.91 [b]	8.41 ± 1.26 [c]
		25	24	273		
		25	15	183		
2	0	25	25	510	100 ± 0 [a]	20.76 ± 0.46 [ab]
		25	25	505		
		25	25	542		
	7	25	25	624	98.67 ± 1.33 [a]	24.6 ± 0.57 [a]
		25	25	634		
		25	24	587		
	14	25	24	582	97.33 ± 1.33 [a]	22.49 ± 1.36 [a]
		25	25	609		
		25	24	496		
	21	25	25	507	97.33 ± 2.67 [a]	17.08 ± 2.77 [b]
		25	25	485		
		25	23	289		
	28	25	17	214	73.33 ± 5.33 [b]	10.17 ± 1.29 [c]
		25	17	231		
		25	21	318		
3	0	25	23	530	56 ±18.90 [bc]	9.81 ± 5.70 [ab]
		25	12	117		
		25	7	89		
	7	25	12	98	76 ±14.05 [abc]	12.77 ± 4.52 [ab]
		25	23	469		
		25	22	391		
	14	25	23	464	93.33 ± 1.33 [a]	20.33 ± 1.62 [a]
		25	24	472		
		25	23	589		
	21	25	21	324	86.67 ± 1.33 [ab]	18.49 ± 2.93 [a]
		25	22	490		
		25	22	573		
	28	25	10	154	42.67 ± 1.33 [c]	4.99 ± 0.64 [b]
		25	11	99		
		25	11	121		

Values represent means ± standard error. a,b,c letters indicate statistically significant differences within treatments (Duncan's significant difference test, $p<0.05$).
